# Gut microbiota and epigenetic inheritance: implications for the development of IBD

**DOI:** 10.1080/19490976.2025.2490207

**Published:** 2025-04-11

**Authors:** Xue Guo, Jianhong Li, Jing Xu, Leiting Zhang, Chen Huang, Yuqiang Nie, Youlian Zhou

**Affiliations:** a Department of Gastroenterology and Hepatology, The Second Affiliated Hospital, School of Medicine, South China University of Technology, Guangzhou, Guangdong, China; b Department of Gastroenterology and Hepatology, Guangzhou First People’s Hospital, School of Medicine, South China University of Technology, Guangzhou, Guangdong, China

**Keywords:** IBD, epigenetics, gut microbes, enviroment, diet

## Abstract

Inflammatory bowel disease (IBD), including Crohn’s disease and ulcerative colitis, is considered significant global health concerns worldwide. Many studies have demonstrated that environmental and dietary factors influence the gut microbiota, which in turn orchestrates the host immune responses. These interactions are also involved in complex metabolic processes that contribute to the pathogenesis of IBD. Furthermore, recent studies in genomics and metabolomics have unveiled the intricate relationship between microbial influencers and host epigenetics. The dynamics of gut microbiota and its metabolites intricately align with DNA methylation, histone methylation, lactylation, glycosylation, and non-coding RNAs, which are key players in epigenetics. Here, we summarize and discuss the complex interplay among gut microbiota, epigenetics, and environmental and dietary factors, and their impact on the pathogenesis of IBD. Furthermore, we highlight the importance of multi-omics technologies in dissecting the host-microbe interactions in IBD, potentially offering a framework for developing effective treatment strategies.

## Introduction

Inflammatory bowel disease (IBD) is a group of chronic conditions, including Crohn’s disease (CD) and ulcerative colitis (UC), characterized by clinical manifestations, including abdominal pain, diarrhea, hematochezia, anemia, and weight loss. Since the beginning of the 21st century, IBD prevalence has remained elevated in Western countries, while its incidence has surged in newly industrialized countries in Asia, Africa, and South America, emerging as a significant global public health challenge.^1^ The latest findings show that^2^ IBD prevalence in England increased from 384.3 in 2015 to 538.7 per 100,000 in 2020, with a forecast of rise to 742.5 per 100,000 by 2027. Research in recent decades has highlighted the importance of the microbiota in normal gut development and maintenance, including digestion, nutrient uptake, metabolism, tissue development, and immunity.^3,4^ Importantly, changes in the composition and abundance of gut microbiota are closely linked to the progression and prognosis of IBD, with intestinal epithelial cells (IECs) responding to resident microorganisms and their metabolites, with microbial signaling being equally important in the development and maintenance of innate and adaptive immune cells.^5,6^ Besides regulating local intestinal cells, gut microbiota can also influence peripheral tissues via the systemic transport of microbiota components and microbiota-derived metabolites.^7,8^ Gut microbiota is believed to be involved in IBD through 3 major pathways – altering the host mucosal barrier, influencing host cellular immune function, and metabolizing dietary factors and host-derived products.^9^ In recent years, advancements in metagenomics and metabolomics technology^8,10^ have substantiated the health-promoting efficacy of probiotics, correlating it with specific genes or individual nucleotides; however, the precise control mechanisms remain inadequately understood. Data from reported studies suggest that^11^ gut microbiota and its metabolites can interact with host epigenetic modifications to manipulate the host chromatin state and function.

Epigenetics is the mechanism by which mammalian cells adapt their transcriptional programs according to their environment. It refers to heritable changes in the function of a gene without altering its DNA sequence, ultimately leading to phenotype change.^12^ Epigenetic mechanisms, including histone modifications and DNA methylation (DNAm), are frequently involved in normal development and disease pathogenesis. Recent studies have found that epigenetics regulates communication between gut microbiota and the IEC genome and plays a key role in the complex pathogenesis of IBD.^13,14^ The significant variation in DNAm and histone modifications constitutes an IBD risk locus, potentially linking epigenetic traits to susceptibility risk loci.^15^

Overall, the gut microbiota and its metabolites profoundly affect host chromatin at DNAm and histone post-translational modification (PTM) levels.^16,17^ In addition to well-established bacterial sensing pathways, microbial signaling is integrated through epigenetic modifications that calibrate the host cell’s transcriptional program without altering the underlying genetic code.^14^ Genome-wide association studies (GWAS) have identified 163 loci associated with susceptibility to IBD, predominantly to CD and UC. Although the findings of this report surpass the complexity observed in other diseases, the extensive number of loci accounts for only a minor proportion of the variability in IBD risk.^18,19^ Monozygotic twin studies have demonstrated a concordance rate of less than 50% in paired IBD, suggesting that the contribution of genomic heritability to IBD is limited.^20^ Therefore, it is widely believed that environmental factors significantly influence the onset and progression of IBD.

Environmental and dietary factors influence the composition and dynamics of gut microbiota, which can trigger phenotypic changes through gene expression without altering the gene sequence. From a nutriepigenetic perspective, dietary factors impact disease progression by influencing epigenetic modifications, which are considered to be the result of interactions among environmental, immune, and genetic factors.^21^ It is noteworthy that the incidence rates of IBD follow global trends associated with lifestyle, industrialization, and Western diets.^22^ Among the reported diet-related risk factors, ultra-processed foods, additives, and emulsifiers are frequently cited,^23,24^ as these can reduce bacterial diversity and increase intestinal permeability, thereby triggering inflammatory mechanisms.^25^ It is particularly necessary to interpret the interaction between the host’s mucosal immune system and the microbes on the epithelial cell surface and within the intestinal lumen from an epigenetic perspective.

**Figure 1.**
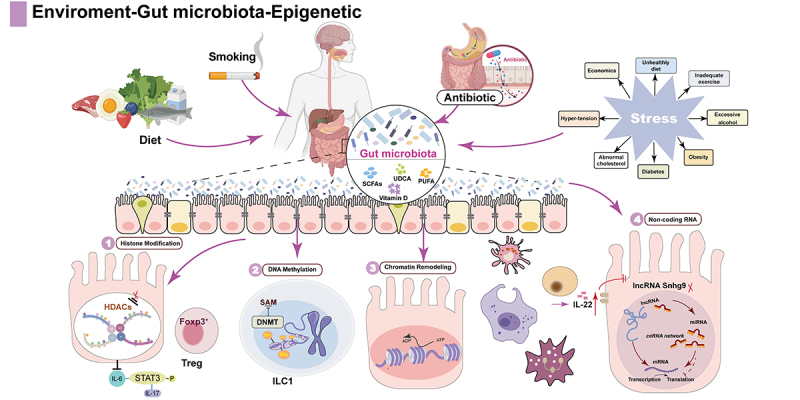
Environmental factors influence epigenetic modifications through gut microbiota and its metabolites in IBD.

This review presents recent studies exploring the complex interactions between environment and diet, gut microbiota, epigenetic modifications, and IBD (Figure 1). Due to the complexity and multifactorial nature of IBD’s pathogenesis, the specific mechanisms underlying its interaction with human gut microbiota and epigenetic regulation are not fully understood. Nonetheless, we aim to provide a concise and informative overview of the current findings in this field.

Environmental factors such as smoking, diet, antibiotics, and stress can influence the composition of gut microbiota and its metabolites, thereby modulating the synthesis of donors and the activity of key enzymes involved in epigenetic modifications. This leads to alterations in intestinal barrier function and immune responses. Epigenetic changes sensitive to the microbiome include: (1) Histone modifications; (2) DNAm; (3) Chromatin remodeling; (4) Non-coding RNAs.

## Epigenetics and gut microbiota

During the development of molecular research in IBD, the DNA methyltransferases (DNMTs), DNA methyltransferases 3a (DNMT3a) and DNA methyltransferases 3b, were identified as key epigenetic regulatory enzymes by GWAS,^18,26^ prompting an exploration of epigenetics rooted in genetics. The epigenetics is considered stable and malleable.^27^ The eukaryotic cell chromosomes are the main carriers of genetic material; nucleosomes, composed of DNA sequences and histones, are the basic units of chromosomes. A 147 bp DNA sequence is coiled around a histone octamer consisting of four histone proteins: H2A, H2B, H3, and H4. These proteins interact with DNA in a non-sequence-specific manner and facilitate the organization, replication, and transcription of genetic information within the DNA molecule. The current research areas in epigenetics include DNAm, histone modification, ncRNAs regulation, and the three-dimensional structural reconstruction of chromatin.^28^ DNAm and histone modifications constitute dynamic regulatory mechanisms of chromatin accessibility, influenced by opposing activities of epigenetic modifying enzymes. Additionally, ncRNAs regulation and the three-dimensional restructuring of chromatin play roles in the initiation and maintenance of epigenetic changes.^29^ Together, these epigenetic mechanisms influence cellular transcription programs by directing histone modifications, transcription factors, and transcription/translation mechanisms with the accessibility of DNA or RNA transcripts.

### DNAm

DNAm is an epigenetic modification process that affects gene expression by adding methyl groups to the cytosine of DNA through DNMTs, which hinders transcription factors from entering the promoter region, thereby inhibiting the expression of the relative protein.^30^ DNAm requires a myriad of raw materials, such as methyl donors, and DNMTs can add one methyl group of the donor S-adenosylmethionine (SAM) to the C-5 position of the cytosine base to produce 5-methylcytosine (DNAm). This process can be reversed by enzymes of the TET dioxygenase family by oxidizing 5-methylcytosine to 5-hydroxymethylcytosine (DNA demethylation).^31,32^ In general, DNAm silences genes, which hinders protein synthesis, whereas DNA demethylation helps synthesize proteins.^33^ DNAm is essential for normal development, as it significantly influences multiple critical processes and serves as a primary regulator of specific gene expression in tissues, ensuring that genes are accurately expressed in the appropriate organ or cell type. However, when DNAm is abnormally high, it can cause many diseases.^34^ The colon is recognized as one of the organs most intimately linked with bacterial folate biosynthesis, given that gut microbiota is continuously exposed to locally produced folate. Research into colorectal cancer (CRC) has highlighted a complex relationship between folate status and cancer risk. Both excessive folate intake and folate deficiency have been associated with an increased risk of CRC development.^35^ Studies have shown significant DNAm alterations in peripheral blood or colon tissue in patients with IBD, which are strongly associated with disease progression.^36^ Differential methylation within certain genes, such as Transporter associated with Antigen Processing 1, is associated with changes in disease course over time.^36,37^ Integrating peripheral blood DNAm, genomics, and transcriptomics in patients with UC and CD can link disease subtypes with clinical outcomes.^15,37^ Although the details of how gut microbiota affects DNAm have not been fully elucidated, the possible mechanisms of direct interactions between gut microbiota and its metabolites and genes have been extensively studied.

### Histone modifications

Gut microbiota can also regulate host chromatin through histone PTMs. Specifically, histone structures have a “tail” at the N-terminal end, and histone modifications usually occur on top of these tails, generally in the form of PTMs of proteins, in a covalent manner.^38^ Classical histone modifications include phosphorylation, acetylation, ubiquitylation, methylation, and glycosylation, while novel modifications include lactylation, crotonylation, succinylation, propionylation, and dihydroxyisobutyrylation. Epigenetic modifications are usually induced by a group of enzymes of opposing classes, particularly histone acetylases, deacetylases, methylases, and demethylases, which can be influenced by microbial metabolites and other environmental factors, thereby dynamically modulating enzyme activity.^38–42^ We examine the histological protein modifications of methylation, lactonization, acylation, ubiquitination, and glycosylation concerning the relationship and research advancements of epigenetic modifications with gut microbiota and IBD.

### ncRNAs

ncRNAs play a significant role in epigenetics, and their functions and mechanisms have been widely explored in recent years. They are a class of RNA molecules that do not code for proteins. They include long-chain non-coding RNAs (lncRNAs), microRNAs (miRNAs), ribosomal RNAs (rRNAs), and transfer RNAs (tRNAs). These ncRNAs are involved in the regulation of gene expression through various mechanisms, including transcription, post-transcriptional level, and epigenetic modifications.

### Chromatin remodeling

Chromatin remodeling regulates chromatin structure through a series of regulatory factors, such as transcription factors, histone-modifying enzymes, and ATP-dependent remodeling complexes. This process involves altering nucleosome density, nucleosome assembly, and DNA – histone interactions, thereby affecting the state of chromatin and DNA accessibility.^43^

Epigenetic regulation is considered an important mechanism through which microorganisms affect host physiology, potentially occurring via several mechanisms: (1) modifying microbial biosynthesis or metabolism by impacting DNA or histone-modified donors; (2) modulating the expression and/or activity of epigenetically modified enzymes; or (3) initiating host-cell intrinsic processes that guide epigenetic pathways. The gut microbiota regulates gut physiology by altering the expression of host genes throughout the gut. Microbiome-sensitive epigenetic modifications encompass DNAm, influenced by short-chain fatty acids (SCFAs), polyphenols, and polyamine metabolites that modulate the activity of DNMTs.^44^ Additionally, histone modifications occur through the mutual regulation of opposing enzymes, which add different chemical groups (e.g., acetyl, methyl, phosphate, ubiquitin) to specific amino acid residues in the histone tail without altering the DNA sequence,^45^ alongside the regulation of ncRNAs. Currently, a significant portion of epigenetic research in humans concentrates on cancer studies,^46,47^ and innovative diagnostic tools aimed at epigenetic biomarkers have transformed cancer diagnosis, positioning proteins that regulate the epigenome as highly promising targets for drug discovery.^48,49^ However, our research on diseases caused by epigenetic enzymes or chromatin alterations due to multiple factors, including IBD, is not clear. Recent reports suggest that gut microbes and their metabolites exhibit great potential in influencing epigenetic modifications, utilizing data from precision medicine biomarker studies involving epigenetic mechanisms to further investigate the specific mechanisms related to IBD with/without therapeutic response, recovery, and potential drug target identification.^11^ Hence, a systematic review and an analysis of the links between gut microbiota and epigenetics are necessary for the research on IBD.

## Impact of environmental factors on gut microbiota and its metabolites

Increasing epidemiological evidence suggests that environmental factors (e.g., urbanization, smoking, air pollution, diet, antibiotics, etc.) play an important role in the pathogenesis of IBD.^50^ For instance, smoking can modulate the composition of the human gut microbiota, and profound changes in its composition occur before and after smoking cessation. These changes are primarily characterized by an increase in the microbial diversity following smoking cessation, along with increased proportions of Firmicutes and Actinobacteria, while the proportions of Bacteroidetes and Proteobacteria decrease.^51,52^ Mice exposed to cigarette smoke exhibited differences in bacterial abundance compared to the smoke-free control group. This included a reduction in *Lactobacillus spp*., an increase in bile acids metabolites, particularly taurodeoxycholic acid in the colon, enhancement of the carcinogenic signaling pathway MAPK/ERK, and compromised gut barrier function.^53^ Diet is a critical environmental factor in the development of IBD and is a focal point of discussion in this article (Table 1). The role of diet has been established both as a risk factor for the pathogenesis of IBD and as a therapeutic approach for the disease.^70^ David et al.^67^ investigated whether human dietary interventions could alter the gut microbiota by dividing participants into two groups based on the type of food they received: a “plant-based diet group” and an “animal-based diet group”. They found that a short-term dietary change over five days was sufficient to reshape the gut microbiota, leading to alterations in the microbial composition and gene activity. Mediterranean, Indian, Japanese, and Southeast Asian diets are rich in unsaturated fatty acids and fiber, which possess anti-inflammatory properties beneficial to IBD patients.^70^ In contrast, a Western high-fat diet, rich in saturated fatty acids but deficient in sugars and fiber, exerts negative regulation on the gut microbiome, reducing the abundance of microorganisms that promote gut barrier repair, such as *Bifidobacterium*, *Lactobacillus*, and *Akkermansia muciniphila*. Such diets can exacerbate gut inflammation by influencing the gut microbiota or activating innate immune receptors and associated cellular stress signaling pathways, thus affecting mucosal integrity and the immune system.^71^ The latest research evaluated the effects of different dietary patterns on the gut microbiota of patients with CD through metaproteomic analysis. It was found that a low – fat and high – fiber diet alters the function of in patients with CD and can enhance the activities of carbohydrate metabolism and short – chain fatty acid synthesis pathways.^72^

**Table 1.** Relationship between metabolites, IBD, and epigenetics.

Categories of metabolites produced by the gut microbiota	Food source	Related gut microbiota	Metabolites	mechanisms that aggravate/relieve UC/CD	Mechanisms regulating epigenetics	References cited
Metabolites produced by gut bacteria from dietary components	Fruits, vegetables, cereals, legumes	Bacteroides，Firmicutes	Polyphenol	Anti-inflammatory and immunomodulatory	Influence on modifying enzyme activity	^54–56^
	High protein diet	*Anaerostipes*, *Bacteroides*, *Clostridium*, *Bifidobacterium*, and *Lactobacillus*	Indole and its derivatives	Protecting barrier integrity and regulating toxicity	Influence on modifying enzyme activity	^57–59^
	Dietary fiber	*Ruminococcus*	SCFA	Maintaining the integrity of the intestinal barrier, regulating immunity and exerting anti-inflammatory effects	Influence enzyme activity and donor synthesis	^60^
	Fish, nuts and seeds, vegetable oils fish oils and soy derivatives	*Alistipes indistinctus,Faecalibacterium prausnitzii*	ω-6 PUFAs	Promotes inflammation levels	Influence on modifying enzyme activity	^61–63^
Metabolites produced by the host and modified by gut bacteria	Cholesterol-rich foods	*Clostridium* spp,Ruminococcaceae	Secondary bile acids Deoxycholic acid (DCA), Lithocholic acid (LCA))	Modulates intestinal inflammation and immunity	Influence on modifying enzyme activity	^64–66^
Metabolites that gut bacteria can resynthesize	Vegetables, Probiotics	*Latilactobacillus sakei*，*Lactobacillus* and *Bifidobacterium*	Vitamins (folic acid)	Reduced inflammatory expression	Influence on donor synthesis	^67–69^

Dorrestein posits that metabolite diversity might even surpass microbial diversity.^73^ Complex phytochemicals in high-fiber foods can be metabolized by gut microbiota into SCFAs, including acetate, butyrate, propionate, and polyphenolic compounds. These metabolites can interact with human intestinal epithelial cells, potentially modulating gene expression through epigenetic modifications.

When discussing UC and CD, the intake of omega-6 (ω-6) polyunsaturated fatty acids (PUFAs), sucrose, monosaccharides, and prolonged fast-food diets is positively correlated with CD severity,^74,75^ whereas breastfeeding, dietary fiber, and fruit consumption may offer protective effects.^76^ For UC, excessive consumption of animal fats and cholesterol, sucrose, and linoleic acid represents high-risk factors, while breastfeeding and a high intake of omega-3 (ω-3) PUFAs, along with vegetable and fruit consumption, can reduce the risk of UC.^77,78^

Current studies from different regions exhibit variability. The American Gastroenterological Association clinical practice guidelines explicitly state^70^ that a healthy, balanced Mediterranean diet, rich in various fruits and vegetables and reduced intake of ultra-processed foods, is associated with a lower risk of developing IBD. A diet low in red meat and processed meats may decrease UC flare-ups but has not been found to reduce CD relapse.^61^ Bolte et al.^79^ investigated 173 dietary factors and 1,425 individual microbes using shotgun metagenomic sequencing to analyze gut microbial composition and function. They discovered that a diet rich in vegetables, legumes, grains, nuts, and fish, with plant-based food intake exceeding animal-based food, could regulate the gut microbiota, possibly preventing gut inflammation processes central to many chronic diseases. Moreover, ω-3 PUFAs and polyphenols can be used to enhance the abundance of SCFA producers.

In existing studies,^80,81^ researchers have investigated the impact of experimental animals consuming single, specific nutrients or additives, leveraging gut microbial metabolites to participate in related immune or stress responses, thereby improving or exacerbating experimental colitis. This approach helps identify specific dietary factors that control gut inflammation and provides insights into how diet influences gut health in genetically susceptible hosts. However, the relevance and consistency of these findings with real-world human diseases remain a gap. Therefore, establishing definitive answers on how diet affects IBD through the gut microbiota and metabolites requires large-scale cohorts, high-throughput multi-omics platforms, advanced statistical analyses, and translational efforts to demonstrate the direct role of nutrients during intestinal inflammation in patients with IBD.

## Metabolites of gut microbiota in epigenetics

Epigenetic regulation mediated by gut microbiota has been established as a form of host-microbe interaction. Broadly speaking, the gut metabolite profile is shaped by diet, modified human-derived metabolites, and microbially-derived compounds, which collectively determine the interactions between the microbes and the host.^82^ Microbial metabolites serve as substrates and cofactors for key host epigenetic enzymes, thereby mediating the link between epigenetic changes and the gut microbiota.^68^ The activity of most histone-modifying enzymes depends on the adequate levels of intermediate metabolites, vitamins, and minerals. These small molecules function as essential substrates, activators, or inhibitors, thus coupling cellular metabolic states to chromatin regulation. In 2017, an integrated approach combining targeted and untargeted human fecal metabolomics was employed. This combined methodology achieves complementarity, providing a more comprehensive analysis of metabolites and playing a crucial role in studying the gut microbiota and its relationship with host health.^83^ By integrating these two approaches, researchers can achieve a deeper understanding of the complex interplay between dietary intake, microbial metabolism, and host epigenetics, enhancing our insights into the mechanisms underlying health and disease. Many gut microbiota -derived metabolites have been identified, which can be broadly categorized into three groups based on their sources and synthesis: (1) metabolites produced by gut bacteria from dietary components, (2) metabolites produced by the host and modified by gut bacteria, and (3) metabolites that gut bacteria can resynthesize. Specific classes of metabolites, especially bile acids, SCFAs, and tryptophan metabolites, can serve as epigenetic substrates, cofactors, or modifiers of epigenetic enzyme activity. We summarize the associations between extensively studied metabolites, IBD, and epigenetics in Table 1, which outlines key findings and their implications.

### SCFA

**Figure 2.**
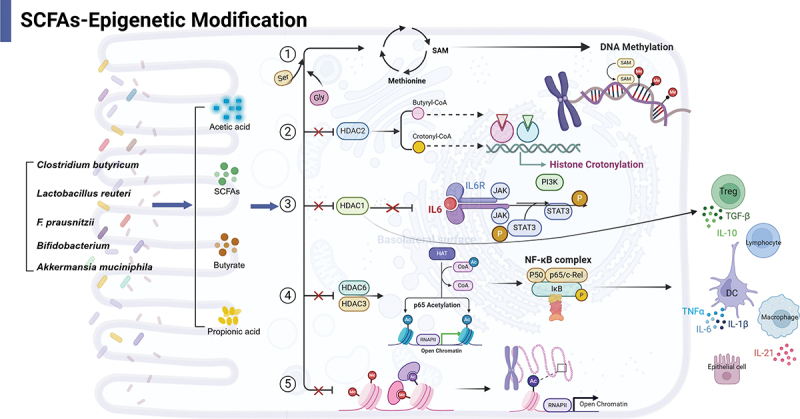
Mechanisms of epigenetic modifications by SCFAs in IBD.

SCFAs are produced by colonic anaerobes fermenting undigested and unabsorbed carbohydrates or glycoproteins, represent the most extensively studied and well-characterized metabolites to date (Figure 2). Dysbiosis of the gut microbiota in patients with IBD is often associated with a decrease in the number of SFCA-producing bacteria, such as *Faecalibacterium prausnitzii* and *Lactobacillus reuter*.^84^ A decline in SCFAs can lead to disruption of the physical and chemical barriers, a decrease in mucus production by tuft and goblet cells, and disturbances in the immune system. One mechanism through which SCFAs exert protective effects is by inhibiting histone deacetylases (HDACs), resulting in increased histone acetylation and modulating the levels of methyl and acetyl donors, thus impacting the progression of IBD.^60^ Butyrate has been shown to promote the development of regulatory T cells (Tregs) and inhibit inflammation in the intestine,^85,86^ which has been partly linked with UDAC activity inhibition at the Foxp3 site by butyrate, thereby promoting histone methylation and Foxp3 development and expression.

SCFAs, such as acetate, propionate, and butyrate produced by various probiotics, modulate epigenetic modifications via multiple pathways: (1) Influence methyl donors by affecting amino acids related to the methionine cycle (e.g., serine, glycine), thus regulating DNAm. (2) Inhibit HDAC2 enzyme activity, increasing the production of butyryl-CoA and crotonyl-CoA, promoting histone crotonylation in colonocytes, and modulating gut homeostasis. (3) Suppress HDAC1 enzyme activity, downregulating the pro-inflammatory IL-6-STAT3-IL-17 signaling pathway, and regulating Treg cells. (4) Inhibit HDAC3 and HDAC6, enhancing p65 acetylation, leading to differential recruitment of NF-κB to pro-inflammatory gene promoters in vitro and in vivo, driving monocytes into a macrophage differentiation program, thereby influencing gene expression in intestinal macrophages, epithelial cells, dendritic cells, and lymphocytes, especially Treg cells. (5) Impact histone acetylation, which is generally associated with chromatin open states and promotes gene transcription. Abbreviations: CoA: coenzyme A; SCFAs: Short-chain fatty acids; HDAC: Histone deacetylase; Treg cells: Regulatory T cells; NF-κB: Nuclear factor kappa-light-chain-enhancer of activated B cells; STAT3: Signal transducer and activator of transcription 3; IL: Interleukin. Created in BioRender. Li, j. (2025) https://BioRender.com/o19u289.

### Micronutrients and minerals

Vitamin D regulates the inflammatory response and maintains intestinal homeostasis, while also participating in epigenetic regulation in the host; a deficiency in vitamin D may result in heightened intestinal permeability and bacterial translocation.^87,88^ Studies have shown that miRNAs mediate intestinal epithelial vitamin D and intestinal inflammation.^89^

**Figure 3.**
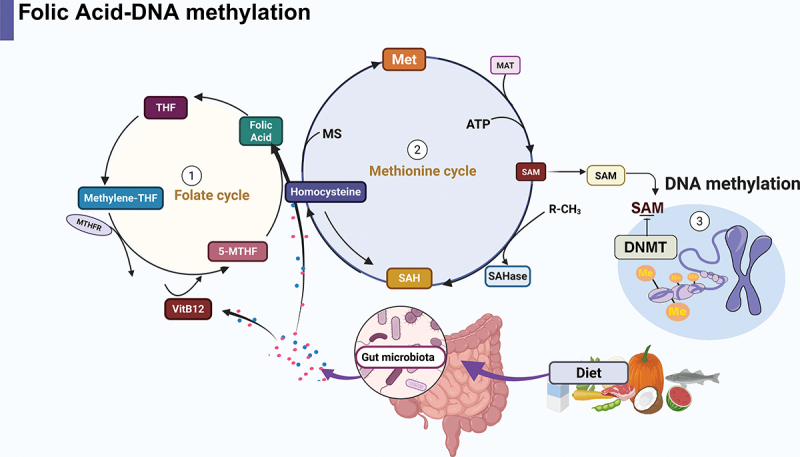
Mechanism of folate-induced DNAm in IBD.

One-carbon metabolism pathways play a crucial role in the activation of immune cells in IBD.^90^ Folate, an essential water-soluble vitamin B that is essentially obtained through diet, serves as a critical donor for DNA synthesis and DNAm. The gut microbiota plays a significant role in folate synthesis and utilization.^67^ *Lactobacilli* and *Bifidobacteria* synthesize folate, thereby increasing DNAm and N6-methyladenosine modification of mRNA in the gut, which is essential for maintaining normal intestinal development.^68^ A folate-deficit diet leads to genomic DNA hypomethylation, affecting DNA stability and gene expression, thereby increasing the risk of tumor formation. Folic acid supplementation can reverse this phenomenon in patients with colorectal adenocarcinoma.^91^ Threonine, serine, and glycine can participate in the folate cycle to initiate the one-carbon metabolic pathway and promote the production of SAM in the methionine cycle,^92,93^ which is a methyl donor for lysine and arginine histone methyltransferases and the DNMT complex^94^ (Figure 3). Similarly, vitamins B3 and B5 regulate deacetylase activity, thereby influencing histone acetylation.^95^ Gut microbiota-generated B vitamins may promote histone and DNAm by affecting the availability of SAM, and can also modulate histone acetylation. The effect of iron on the intestinal epigenome is not yet known.^96^ However, reduced iron concentrations in gut microbiota were positively correlated with butyrate and propionate reduction.^97^ Another study^96^ demonstrated that chronic iron exposure in colonic cells leads to significant hypomethylation of the epigenome. Iron-mediated epigenetic modifications may occur in iron-rich intestinal epithelial cells, potentially influencing DNAm patterns and cellular function.

Folate from dietary sources participates in the process of DNAm through complex folate and methionine cycles, which are tightly interconnected. (1) Folate Cycle: Dietary folate is enzymatically converted to THF in the small intestine. THF accepts one-carbon units within cells to form various active folate forms. Among these, 5-methyl-THF serves as the methyl donor for remethylation of homocysteine. (2) Methionine Cycle: 5-methyl-THF acts as a methyl donor with vitamin B12 (cobalamin) as a cofactor. Homocysteine is methylated by MS to regenerate methionine. The newly formed methionine then combines with ATP under the action of MAT to produce SAM. (3) DNAm: Under the DNMTs, SAM donates methyl groups to specific cytosine residues, completing the process of DNAm. Abbreviations: THF: Tetrahydrofolate; 5-methyl-THF: 5-Methyltetrahydrofolate; MS: Methionine synthase; MAT: Methionine adenosyltransferase; SAM: S-adenosylmethionine; DNMTs: DNA methyltransferases; DNAm: DNA methylation; IBD: Inflammatory bowel disease. Created in BioRender. Li, j. (2025) https://BioRender.com/l34n379.

### Polyphenol

**Figure 4.**
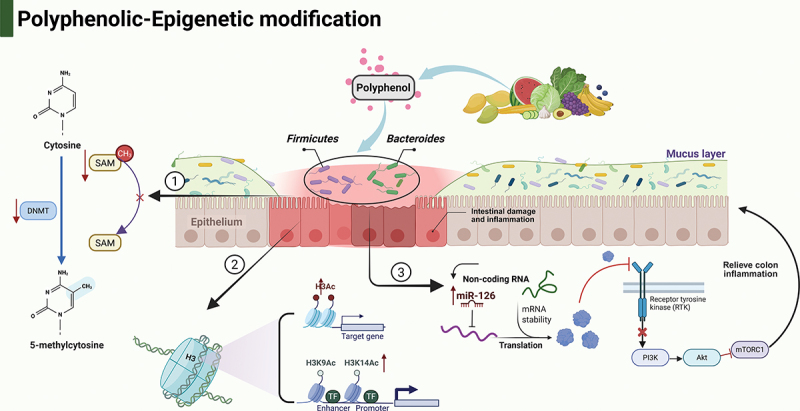
Polyphenols influence ncRNA in IBD.

Polyphenolic compounds are primarily synthesized in fruits, vegetables, and other plant sources. Over 90% of unabsorbed polyphenols in the colon can be metabolized and transformed into various SCFA derivatives by the gut microbiota,^98^ contributing to microbial homeostasis and alleviating IBD. Polyphenols inhibit DNAm and DNMT activity in two ways: by decreasing intracellular SAM concentrations and through noncompetitive inhibition of DNMT activity or by intercalating into DNMT binding clefts to form competitive inhibition. For example, Polyphenolic compounds such as curcumin can effectively inhibit the production of reactive oxygen species by covalently blocking the catalytic cysteine C1226 of DNMT1, transferring electrons to free radicals, and simultaneously activating antioxidant enzymes, thereby ameliorating oxidative stress and inflammation.^54,55^ Epigallocatechin-3-gallate, the predominant polyphenol in green tea, acts as an inhibitor of DNMTs and enhances histone acetylation, particularly at H3K9/14ac and H3ac sites.^99^ Gallic acid, the most abundant polyphenol in mangoes, alleviates inflammatory responses in mice by upregulating miRNA-126 expression both in vitro and in vivo, thereby modulating the PI3K/AKT/mTOR signaling pathway^100^ (Figure 4).

Polyphenolic compounds from fruits and vegetables can influence epigenetic modifications through multiple pathways: (1) Decreasing intracellular SAM concentrations and non-competitively inhibiting DNMT activity or competitively inhibiting DNMT by inserting into DNMT-binding pockets, thereby regulating DNAm; (2) Increasing histone acetyltransferase (H3K9/14ac and H3ac) activity to regulate histone modifications; (3) Modulating the expression of non-coding RNA miR-126, which subsequently inhibits the PI3K/AKT/mTOR signaling pathway. Abbreviations: SAM: S-adenosylmethionine; DNMT: DNA methyltransferase; H3K9/14ac: Histone H3 lysine 9 and 14 acetylation; H3ac: Histone H3 acetylation; miR-126: MicroRNA-126; PI3K: Phosphoinositide 3-kinase; AKT: Protein kinase B; mTOR: Mammalian target of rapamycin. Created in BioRender. Li, j. (2025) https://BioRender.com/y59j565.

### Bile acids

**Figure 5.**
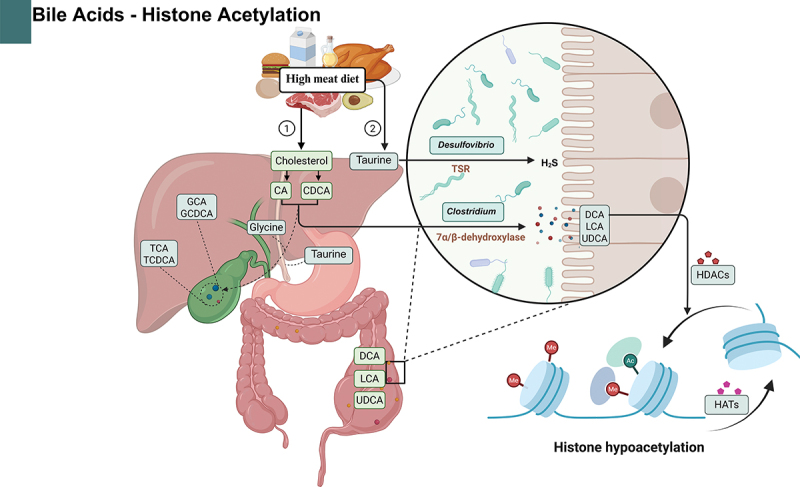
Impact of bile acids on histone modifications in IBD.

A bidirectional relationship exists between bile acids and the microbiota, playing a crucial role in maintaining intestinal barrier function.^101^ Bile acids are derived from cholesterol in food, and the process of 7α/7β-dehydroxylation of bile acids is uniquely observed in anaerobic bacteria within the human colon^65^ (Figure 5). High-performance liquid chromatography-tandem mass spectrometry analysis revealed that compared to healthy individuals, fecal levels of conjugated bile acids were significantly elevated in patients with active IBD, while secondary bile acids levels were reduced.^102^ Studies comparing fecal samples from ileal pouches of patients with UC to those with familial adenomatous polyposis showed decreased levels of lithocholic acid and deoxycholic acid, which correlated with reduced abundance of Ruminococcaceae bacteria.^103^ A high-fat diet promotes the conjugation of bile acids with taurine in the liver, leading to increased abundance of *Bilophila wadsworthia* (a hydrogen sulfide-producing bacterium) in IL10 knockout mice, thereby exacerbating colitis development. Akare et al.^104^ reported that ursodeoxycholic acid regulates chromatin by inducing histone hypoacetylation and promotes differentiation and senescence of colorectal cancer cells.

In IBD, dietary cholesterol from high-meat intake is converted into primary bile acids and taurine in the liver. (1) Primary bile acids, including CA and CDCA, are synthesized by hepatocytes and stored in the gallbladder. In the colon, certain anaerobic bacteria within the Clostridium genus possess 7α-dehydroxylase, which converts primary bile acids into secondary bile acids such as DCA, LCA, and UDCA. These secondary bile acids activate histone HDACs, leading to histone hypoacetylation. Meanwhile, high-meat diets increase taurine intake, which is primarily metabolized in the small intestine by TSR, an enzyme produced by anaerobic bacteria of the Desulfovibrio genus, resulting in H2S production that exacerbates colitis development. Abbreviations: IBD: Inflammatory bowel disease; CA: Cholic acid; CDCA: Chenodeoxycholic acid; DCA: Deoxycholic acid; LCA: Lithocholic acid; UDCA: Ursodeoxycholic acid; HDACs: Histone deacetylases; TSR: Taurine sulfur reductase. Created in BioRender. Li, j. (2025) https://BioRender.com/o19u289.

### Tryptophan

Tryptophan can be metabolized by gut microbiota into a series of indole metabolites. A cohort study involving 535 patients with IBD found that disease activity was negatively correlated with tryptophan levels, with a more pronounced reduction observed in patients with CD compared to those with UC.^105^ *Lactobacillus*-regulated tryptophan metabolite norharman inhibits the enzymatic activity of HDACs, thereby suppressing the activation of M1 macrophages.^58^

Tryptophan and its metabolites exert their effects through the activation of the aryl hydrocarbon receptor (AhR), which plays a critical role in maintaining intestinal homeostasis. Studies have shown that AhR activation modulates the “HDAC3-acetylation” signaling pathway to enhance the degradation of fatty acid synthase, thereby limiting fatty acid synthesis and reducing extracellular matrix deposition, alleviating intestinal fibrosis – a complication associated with CD.

### PUFAs

PUFAs, including ω-3 PUFAs and ω-6 PUFAs, are essential fatty acids primarily derived from dietary sources. Epidemiological evidence indicates an inverse relationship between ω-3 PUFAs intake and both the severity and incidence of CD.^106^ Specifically, a low ratio of ω-3 to ω-6 PUFAs is recognized as a risk factor for CD pathogenesis. Dietary consumption of oxidized PUFAs has been linked to exacerbated colitis severity, while diets rich in non-oxidized PUFAs do not promote colitis.^74^ A large-scale prospective study conducted in Denmark revealed that arachidonic acid, an essential ω-6 PUFAs, is excessively present in the mucosa of patients with UC and in animal models. The concentration of arachidonic acid was found to correlate with the extent of histological inflammation.^107,108^

The influence of gut microbiota and their metabolites on the host’s epigenetics and their potential impact on disease in particular cell types remain largely unexplored. However, it is clear that the precise effects of all these altered metabolites on enzymes involved in epigenetic modifications in IBD could serve as a key mediator between host and microbes in human IBD. This review will focus on how microbes and metabolites affect epigenetic modifications during IBD onset and progression.

## The gut microbiota and its metabolites influence IBD progression through DNAm

Gut microbiota remodels IEC DNAm to control intestinal homeostasis and inflammation. To determine the effect of gut microbiota on host genome-wide DNAm, Ansari et al.^16^ performed Whole-Genome Bisulfite Sequencing (WGBS) on colonic crypt IECs, and data analysis revealed that exposure to the commensal microbiota induces profound epigenetic changes in regulatory elements in low methylated regions. Exposure to microbiota during dextran sodium sulfate (DSS) -induced acute inflammation results in DNAm of regulatory elements and modifications in open chromatin, thereby affecting functional gene expression programs associated with colitis and CRC. Since the upregulated hypomethylation genes were enriched in important functions, such as chemotaxis and inflammatory response of immune cells, it was speculated that changes in gene expression may be caused by epigenetic remodeling of IECs, which demonstrated that microbiota-induced epigenetic remodeling is necessary for maintaining intestinal homeostasis in vivo. Similarly, DNAm plays an important role in the development of sporadic CRC, and although IBD and CRC are two different diseases, patients with IBD have an increased risk of CRC.^109^ Pekow et al.^110^ identified variations in DNAm and gene expression between sporadic CRC and IBD-associated CRC, noting that DNAm modulates the transcription of critical genes implicated in CRC progression. This may elucidate the disparities in clinical presentation and outcomes between IBD and sporadic CRC. Additionally, Zhang et al.^111^ systematically demonstrated that the gut microbiota diminishes bile acid levels in the intestine and regulates the DNA hydroxymethylation of innate lymphoid cells and their progenitors, thereby facilitating the differentiation of Innate Lymphoid Cell 1, which is crucial for the development of the early mucosal immune system. gut microbiota also regulates DNAm in Tregs by increasing the expression of the DNAm junction protein, Ubiquitin-like with PHD and Ring Finger Domains 1 (Uhrf1). Uhrf1 forms a complex with DNMT1 and HDAC1, and T cell-specific knockdown of Uhrf1 impairs the expression of normal cell-cycle genes in Tregs, which can lead to spontaneous colitis.^112^ Clinical studies have shown that exposure to probiotics or pathogenic bacteria causes different patterns of DNA modification in fetal and adult epithelial cells, potentially making them susceptible to or protected from various diseases.^113^

Gut microbiota and its metabolites can provide methyl donors. It has been found that a widespread reduction in the production of one-carbon compounds by gut microbiota metabolites has been associated with defects in the generation of DNAm.^114^ Folic acid can provide methyl groups for DNAm^115^ and gut commensal microbes can metabolize the dietary methionine to SAM for its use as a primary substrate during DNAm. Thus, changes in the composition of the gut microbiota can affect SAM availability and alter the state of DNAm in the host. Liu et al. ^116^ found significant changes in the DNA methylome of CRC tissues compared to neighboring normal tissues by combining macrogenomics, 16S rRNA, and WGBS analyses. Substantial changes in microbial-derived methyl donor-associated pathways occurred between tumor and adjacent normal tissues, with significant enrichment of microbial-associated epigenetic loci within the promoter regions of genes in adjacent normal tissues, but absent in tumor tissues. Choline, which maintains the structural integrity of cell membranes and supports cholinergic neurotransmission, is an essential nutrient and methyl donor for epigenetic regulation. Chronic choline deficiency can cause significant alterations in epigenetic regulation, while the gut microbiota is an important regulator of choline bioavailability, and choline-consuming bacteria deplete the metabolites of the methyl donor, thus affecting physiological functions.^114,117^

Gut microbiota and their metabolites can also be involved in the process of methylation or demethylation through activation of DNMTs or TET dioxygenases.^118^ Fazio et al.^119^ have reported that genetic variation at the DNMT3a locus has been associated with IBD. IBD plays a key role in maintaining intestinal homeostasis and barrier function. Impaired DNMT3a function results in global DNA hypomethylation, which is potentially linked to the etiology of IBD. Zhang et al.^120^ also reported that DNAm alterations in DNMT3a mediate the effects of smoking on IBD, and that DNMT3A-mediated epigenetic regulation of gene expression is crucial for the formation of precise epithelial junctions, maintenance of intestinal homeostasis, and sustenance of optimal epithelial barrier function. The mechanisms involved may be related to immune responses, alterations in the microbiome, and pro-inflammatory epigenetic modifications.

DNAm is widely involved in biological processes such as cell development, differentiation, and tissue homeostasis.^15^ Impaired DNAm regulation causes inflammation in many diseases, including IBD.^121,122^ Nonetheless, the comprehension of DNAm defects in chronic inflammation remains inadequate; however, current findings suggest a plausible hypothesis that variations in methylation levels correlate with gut microbiota and that their interactions influence the onset and progression of IBD, although further research is required.

## The gut microbiota and its metabolites influence IBD progression through histone modifications

### Advances in histone methylation in IBD

Histone methylation can occur at different histone sites, primarily at lysine and arginine residues, and can be controlled by multiple positive and negative regulators, which can activate or repress transcription at a single site.^123^ Distinct enzymes govern histone modifications: chromatin ‘writers’ (methyltransferases) that facilitate regulatory modifications; ‘erasers’ (demethylases) that eliminate modifications; and ‘readers’ that identify modifications and affect gene expression. Collectively, these enzymes significantly contribute to gene activation and repression processes.^123^ Histone H3 serves as a principal locus for histone methylation, with the methylation of the fourth lysine on histone H3 (H3K4me1–3) being among the most extensively examined histone modifications due to its strong association with the active state of gene expression and the dysregulated enzymes often linked to its modulation in cancer research.^124,125^ Its methyltransferase and demethylase activities dynamically regulate H3K4 methylation levels. Researchers^126^ analyzed histone methylation profiles in ileal epithelial cells from pediatric patients with IBD that are sensitive to gut microbiota colonization and found that microbiota-dependent H3K4me3 identifies targets that undergo epigenetic modifications due to gut microbiota exposure during IBD flare-ups, which can be correlated to the composition of the unique microbiota in IBD and epigenetic dysregulation of the H3K4me3 locus region. The level of intestinal methylation in mice is closely linked to microorganisms. Krautkramer et al.^127^ investigated the histone acetylation and methylation profiles in the proximal colon of germ-free mice, conventionally raised (ConvR) mice, and conventionalized (ConvD) mice. They discovered colonization led to a significant increase in H4 acetylation levels across all three mouse groups, with a more pronounced effect observed in ConvD mice. H3K27 methylation was also significantly elevated, indicating that gut microbiota influences the acetylation and methylation of host tissues, thereby suggesting that gut microbiota is pivotal in regulating chromatin in host tissues. Methyltransferase Nsun2-mediated m5C modification is essential for Th17 cell homeostasis and contributes to the pathogenesis of colitis in mice, and Nsun2 deficiency leads to amelioration of the progression of Th17 cell-induced colitis.^128^

### Advances in histone lactylation in IBD

Histone lactation is a novel type of PTM discovered by in 2019 by Professor Zhao.^42^ Lactic acid accumulated during metabolism has been identified as a major carbon source for the mitochondrial tricarboxylic acid cycle in normal tissues and tumors^129,130^ and also as a precursor that promotes lactate modification of histone lysine, regulates gene transcription, and participates in the homeostatic regulation of bacterial-infected M1 macrophages, thereby modulating inflammation, cancer, and other diseases.^42,131^ Histone lactation is not only an epigenetic modification, but it is also closely related to cellular metabolism. Lactate produced by glycolysis is a substrate for histone lactylation, which affects gene expression through metabolic pathways.^132,133^ Macrophages can swiftly react to microbial ligands and external stimuli, gradually shifting from a pro-inflammatory to a reparative state, a transition process critical for anti-inflammation and homeostasis restoration. Irizarry et al.^134^ identified B-cell adapter for PI3K as a key switch in the intracellular regulation of this process, which, by connecting with Toll-like receptor (TLR), enhances glycolysis and lactate production, thereby facilitating histone lactylation, which subsequently reprograms macrophage metabolism and regulates the shift from inflammatory to reparative macrophages. Sun et al.^135^ also explored the effect of lactate in colitis pathogenesis on the regulation of macrophage transcriptional levels. They discovered that lactic acid intervention elevated histone H3K9 acetylation and histone H3K18 lactylation levels in BMDM, hypothesizing that lactic acid’s inhibitory effect on colitis may be associated with genes or proteins that modulate acH3K9 and laH3K18. Xie et al.^136^ used techniques, such as histone lactylation histology, to discover that lactation is upregulated in CRC tissues and is associated with poor prognosis. K(lysine) acetyltransferase 8, a pan-lactate transferase, was identified for the first time, which can promote CRC by modifying the lactate of lysine 408 sites of eukaryotic translation elongation factor eEF1A2 to increase the translation efficiency of the protein. IBD is closely related to CRC development, which is a research idea for exploring histone lactylation in IBD.

### Advances in histone acylation in IBD

Histone acylation is a critical area of research in epigenetics. In this process, various acyl groups are introduced to the amino acid residues of histones, thereby affecting gene expression and chromatin structure. These modifications include acetylation, crotonylation, succinylation, malonylation, and so on. The versatility of different histone lysine acylates is determined by their different chemical properties, with each modification possessing a unique function and biological significance.^137^

Histone acylation is sensitive to changes in the commensal microbiota *in vivo*.^138^ Gut microbiota can modify the histone structure through metabolites, including SCFAs, thereby altering host gene expression levels. Previous studies have shown^139^ that microbial-derived metabolites, particularly SCFAs like acetic acid, propionic acid, and butyric acid, significantly influence mucosal and systemic immune responses, and that reduced levels of SCFAs in the gut are directly correlated with increased susceptibility to inflammatory diseases.^140^ In contrast, histones with vast modifications of SFCAs result in histone acylation.^141^

Acetylation is a common histone modification that occurs mainly on lysine residues, increasing the accessibility of DNA and thus promoting gene expression.^142^ gut microbiota can ferment dietary fiber into butyrate to influence host histone acetylation.^143^ Butyrate promotes histone acetylation by inhibiting HDAC, and also it can oxidize histones to acetyl coenzyme A (CoA), an essential histone acetyltransferase cofactor.^144^ Butyrate-derived acetyl CoA induces histone acetylation and regulates gene expression by stimulating histone acetyltransferases (HAT) and inhibiting HDAC in an ATP Citrate Lyase (ACLY) -dependent and ACLY-non-dependent manner, respectively.^145^ HDAC can mediate intestinal homeostasis in IECs by relying on the expression of commensal bacteria, e.g, HDAC3 coordinates commensal microbial-derived signals to maintain a normal host – symbiont relationship.^14^ Butyric acid enhances p65 acetylation by inhibiting HDAC3 and HDAC6 and allowing differential recruitment of NF-κB to pro-inflammatory gene promoters *in vitro* and *in vivo*.^146^ It drives monocytes into the macrophage differentiation program, thereby affecting gene expression in intestinal macrophages,^147,148^ epithelial cells, dendritic cells, and lymphocytes, especially Tregs.^85^ *F. prausnitzii* produces butyric acid to inhibit experimental enteritis in mice. *F. prausnitzii* produces butyric acid to inhibit experimental enteritis in mice, which regulates Tregs by inhibiting HDAC1 and transcriptionally downregulating the pro-inflammatory IL-6/STAT3/IL-17 signaling pathway and concomitantly promoting Foxp3 expression.^149^ In addition to SCFAs, Wu *et al*.^150^ found that inositol trisphosphate, another gut microbiota metabolite, activates mammalian HDAC to promote epithelial repair and ameliorate colitis-mediated intestinal damage. This suggests that HDAC3 can orchestrate intestinal dynamics by sensing different microbial signals resulting from changes in diet or gut microbiota.

Fellows et al.^151^ showed that gut microbiota depletion leads to a decrease in SCFA and that butyrate can act as an inhibitor of HDAC2 by increasing butyryl-CoA and crotonyl-CoA production, thereby facilitating colonic histone crotonylation and maintaining intestinal homeostasis. Furthermore, histone crotonylation can connect chromatin structures to the gut microbiota through HDAC and SCFA. Interestingly, the addition of butyrate or propionate rapidly and consistently promotes histone acetylation, facilitating the rapid conversion of propionate and butyrate to the corresponding acyl-CoA, which may be related to the inhibition of HDAC activity or activation of the acetyltransferase p300, suggesting that the amount of open chromatin can be increased by a low concentration of SCFAs.^147,152^

Gates^138^ identified elevated levels of histone butyrylation and propionylation in the cecum compared to other anatomical regions, aligning with the cecum’s microbial milieu and the prevalence of SCFAs. Conversely, the examination of mice reared in a sterile environment and those administered antibiotics revealed markedly reduced levels of intestinal histone acetylation, butyrylation, and propionylation; however, the administration of glycerol tributyrate effectively restored histone butyrylation and cellular butyryl CoA levels. This study found that gut microbiota and the metabolites can promote histone butyrylation and alter the expression of metabolism-related genes, affecting the progression of IBD.

Cysteine palmitoylation (S-palmitoylation) is a reversible PTM directed by the Asp-His-His-Cys (DHHC) family of palmitoyltransferases and can be reversed by several acyl – protein thioesterases. Zhang et al.^153^ demonstrated^106^ that DHHC7 and acyl protein thioesterase 2 (APT2) catalyze STAT3 and undergo reversible S-palmitoylation, which promotes STAT3 by facilitating membrane binding, phosphorylation, and nuclear translocation to achieve cell differentiation, and when the palmitoylation – depalmitoylation cycle was interrupted by knockdown of DHHC7 or inhibition of APT2, symptoms of colitis were alleviated in a mouse model.

Histone succinylation refers to transferring a succinyl group from a donor (e.g., succinyl CoA) to a lysine residue on a substrate protein, resulting in a covalent bond formed either enzymatically or nonenzymatically. This process is integral to the tricarboxylic acid cycle, glucose metabolism, and various energy metabolism pathways, intricately linked to inflammation, metabolic disorders, and neoplasms. Sirtuin 5 is a mitochondrial protein that facilitates the desuccinylation of the Pyruvate Kinase M2 Isoform (PKM2) metabolic enzyme. Sirtuin 5-mediated desuccinylation of the PKM2 enzyme inhibited LPS-induced IL-1β expression, thereby preventing DSS-induced colitis in mice.^154^

### Advances in histone ubiquitination in IBD

Histone ubiquitination modification is the process of attaching ubiquitin, a small protein, to a target protein through covalent bonds, which works synergistically through ubiquitin-modifying enzymes (UMEs) to coordinate the optimal ubiquitination of target proteins to maintain intestinal homeostasis.^155,156^ Ubiquitination is an important PTM that is regulated by the UMEs.^156^ It consists of ubiquitinylating enzymes and deubiquitinating enzymes (DUBs), including ubiquitin-activating enzymes (E1), ubiquitin-conjugating enzymes (E2), ubiquitin ligases (E3), which progressively attach ubiquitin to target proteins; DUBs regulate the ubiquitin signaling pathway by shortening or removing the ubiquitin chain to reverse histone ubiquitination.^157,158^ Histone ubiquitination is an important PTM in the inflammatory response, affecting the function of key proteins in the signaling pathway.^156^ In recent years, histone ubiquitination has been shown to play a crucial role in the pathogenesis and development of IBD.^18^ Dysregulation of E3 ubiquitin ligase-associated response processes has been reported in IBD and in many immune-related diseases.^159^ Ankyrin repeat and SOCS Box-containing-3, a member of the E3 family, promotes the TLR-Myd88/TRIF/independent NF-κB pathway by specifically catalyzing K48-linked polyubiquitination in IECs of aberrant activation and intestinal microbes imbalance.^160^ Ovarian tumor family deubiquitinase (OTUD) 4 is a multifunctional deubiquitinating enzyme that plays a key regulatory role in infection, DNA damage repair, regulation of RNA processing, and tumors.^161–163^ OTUD4 inhibits the K63 polyubiquitination of MyD88 and the subsequent activation of MyD88-dependent NF-κB and MAPKs, thereby limiting the expression of antimicrobial peptides, influencing the composition of gut microbiota, modulating the intestinal antimicrobial immune response, and exacerbating intestinal inflammation.^164^ Similarly, OTUD6A selectively cleaves the K48-linked polyubiquitin chain from NLRP3 at the K430 and K689 loci to enhance the stabilization of NLRP3, leading to IL-1β levels and inflammation, and exacerbating intestinal inflammation in mice.^165^

### Advances in histone glycosylation in IBD

Glycosylation^166^ is also an important PTM of proteins that are widely involved in the development and progression of tumors, inflammation, and other diseases. Over 50% of intracellular proteins are subject to glycosylation, while epithelial polysaccharides constitute a significant element of the intestinal mucosa, participating in interactions between gut microbiota and IECs, and are modified during infections with pathogenic bacteria and in conditions such as IBD.^167^ Interestingly, the presence of similarly modified serine and threonine residues between other common PTM components of O-GlcNAcylation leads to a wide range of crosstalk, such as phosphorylation, ubiquitination, acetylation, and methylation.^168^ For example, O-GlcNAcylation can inhibit the ubiquitination of target proteins by various mechanisms to prevent their degradation.^169^ O-GlcNAcase has a C-terminal HAT-like and an N-terminal O-GlcNAc hydrolase structural domains, and thus, O-GlcNAcylation and acetylation can regulate each other.^170^

Intestinal epithelial glycosylation spatially regulates intestinal microbes, and alterations in the glycosylation of epithelial cells, including disruptions in mucin-type O-glycans, N-glycans, and terminal glycan structures, result in spatial patterns of ecological dysbiosis in IBD.^171,172^ *N*- and O-conjugated glycans are major components of the glycoproteome, with intestinal epithelial O-glycosylation accounting for 80% of MUC2 composition, the most abundant intestinal mucin spatially regulating gut microbiota; altered glycosylation induces dysbiosis in IBD.^173^ The N-glycosylation of host mucus is crucial for sustaining a specific population of intestinal commensal bacteria, and the expression of genes associated with N-glycosylation is impaired in patients with UC exhibiting diminished expression.^174^ It has been shown that mucoglycan – microbe interactions contribute to the positive selection of commensal microorganisms during slow transport through the colon and support the negative selection of pathogens during rapid transport through the small intestine.^175^

Epithelial glycosylation contributes to gut microbiota homeostasis. Yao et al.^176^ found that mucin sialylation, a glycosylation modification mediated by the sialic acid transfer enzyme ST6GalNac1 (ST6), protects the intestinal mucus barrier and modulates gut microbiota homeostasis, thereby affecting host susceptibility to intestinal inflammation, through a proteomics analysis of N-linked intact glycopeptides. This study demonstrated the critical role of ST6 in the salivary acidification of intestinal proteins and the maintenance of normal physiological processes in IECs. He *et al*.^177^ discovered that N-glycosylated bacterial colonization factor-1 directly interacts with *Escherichia coli* via its bacterial hair protein YdeR, facilitating *E. coli* colonization and that the distribution of the bacterial population significantly influences the progression of IBD.

Epithelial glycosylation participates in barrier formation, host – microbe symbiosis modulation, and immunity. The MUC2 mucus layer can form a single loose layer in the small intestine and an externally loose yet internally adherent layer in the colon. While the loose layer in the small intestine and colon serves as a permeable habitat and nutrient matrix for gut microbiota, the inner mucus layer is impenetrable to bacteria and prevents bacterial – epithelial interactions in the distal intestine.^178,179^ Patients with IBD have altered epithelial cell surface glycosylation, including changes in terminal structures at the O-glycosyl, N-glycosyl, and glycolipid levels, such as increased terminal sialylation, decreased fucosylation, and decreased sulfation. Changes in serum N-glycosylation associated with IBD also occur, including an increase in large glycosyl groups, a decrease in heterozygous high-mannose structures, a decrease in fucosylation and galactosylation, and an increase in sialylation. Alterations in glycosylation occur prior to inflammation, with heightened T antigen expression noted in unaffected homozygous twins, potentially resulting in an augmented inflammatory profile.^180,181^ O-glycans may play two major roles in MUC2 synthesis^180^ by facilitating the tight packing and storage of MUC2, while glycans on MUC2 may prevent bacterial proteases from degrading the mucus. Using a mouse model, it was demonstrated^178^ that glycans are essential for synthesizing the MUC2 mucus layer.

### Advances in histone phosphorylation in IBD

Histone phosphorylation is the process of phosphorylation or dephosphorylation of histone proteins accomplished by the addition of phosphate groups to their tails by protein kinases or the removal of phosphate groups by protein phosphatases. It plays an important role in DNA repair structure, transcription, and chromatin compression during cell division and apoptosis, and is associated with the regulation of gene expression. The Sin3-associated polypeptide p15 peptide was found to target the HDAC5 protein and inhibit its phosphorylation to regulate macrophage polarization and thus exert anti-inflammatory effects in a mouse model of DSS-induced apoptosis.^182^ Researchers in the Netherlands^183^ performed a cluster heatmap analysis of differentially phosphorylated sites in human cells infected with the enterovirus CVB3 and found that the key regulatory kinases, which mediate the process, as well as their mTORC1 signaling pathways, were reduced. The researchers also analyzed genes downstream of the mTORC1 signaling pathway and found that downstream ULK1, P70, 4E-BP, and TFEB phosphorylation levels were also downregulated. Wilson et al.^184^ demonstrated that phosphorylation of STAT3 is a modification required for the formation of Th17 cells and expression of *RORγt*. In the absence of MyD88 or TLR2, histones failed to induce STAT3 phosphorylation, and the rapid phosphorylation of STAT3 following histone addition further suggests a direct correlation between histone binding and STAT3 phosphorylation.

## The gut microbiota and its metabolites influence IBD progression through ncRNAs

### lncRNA

LncRNAs^185^ are a class of ncRNAs with more than 200 nucleotides in length. Multiple studies have found that lncRNAs are involved in various biological processes, including epigenetic regulation, transcriptional regulation, and post-transcriptional regulation.

In IBD, lncRNAs affect intestinal mucosal function by regulating epithelial cell apoptosis, altering intestinal mucosal barrier permeability, and promoting inflammatory responses.^186,187^ It is well known that IBD leads to IEC damage, increased intestinal mucosal inflammation, and entry of bacteria or bacterial products into the intestinal barrier.^188^ In this regard, it is important to emphasize that IBD is a major cause of IEC damage. Epithelial regeneration is a critical step in wound healing in the intestinal mucosa, and studies have shown that IECs express many negative regulators to control IEC regeneration, such as the P53 protein.^189^ In contrast, Geng et al ^190^ found that H19 lncRNA, an IL22-induced inflammatory lncRNA, antagonizes the negative regulators of IEC proliferation, thus playing an important role in maintaining intestinal epithelial regeneration in inflammatory conditions. Ma et al.^186^ linked lncRNA complex traits to human diseases and discovered that lncRNA-C5orf56, an IBD-associated region, interacts with the gut microbiota and controls intestinal inflammation by protecting against IBD. gut microbiota can influence the metabolic and physiological state of the host by regulating specific lncRNAs. Wang et al.^185^ discovered that gut microbiota can alter lipid metabolism in the murine intestine by suppressing the expression of Snhg9, a lncRNA in small intestinal epithelial cells, which holds considerable importance for advancing research on the impact of gut microbiota-induced metabolic disorders on IBD.

### miRNA

Short-stranded ncRNAs (e.g., miRNAs) are a class of ncRNAs with approximately 18–23 nucleotides in length and regulate protein translation by binding to mRNAs and inhibiting their translation or causing their degradation, which in turn affects physiological and pathological processes in the human body.^191^ The interaction between gut microbiota and miRNAs can affect the onset and progression of IBD by modulating multiple aspects of mammalian intestinal homeostasis, including intestinal barrier integrity, microbial homeostasis, injury or pathogen detection, and the activation of innate and adaptive immune responses.^192^

The expression level of miR-31 in patients with IBD is positively correlated with the severity of the disease, and its expression in IECs is closely related to IBD pathogenesis.^193^ Intestinal epithelium-specific deletion of miR-31 exacerbates colitis and hampers the repair of the damaged intestinal epithelium in mice. Moreover, they discovered that miR-31 can enhance the regenerative repair of intestinal epithelium by suppressing inflammatory factor receptors or signaling proteins, thereby negatively modulating the inflammatory response, while simultaneously activating WNT and inhibiting the Hippo signaling pathway. This research result suggests that miR-31 is involved in initiating resistance to inflammation and accelerating self-repair regulatory mechanisms during the IEC-mediated inflammatory response. miR-31 or analogs have the potential to be developed as therapeutic drugs for IBD. Liu et al.^194^ demonstrated that fecal miRNAs can modify the gut microbiota and that mice with gluconic acid-induced colitis lacking Dicer (miRNA processing enzyme) cannot produce miRNAs, resulting in exacerbated symptoms.

Recently, it has been reported^192^ that administration of a miR-423-5p inhibitor to mice with enterocolitis improves the epithelial barrier function, which is associated with targeting of Claudin 5 mRNA miR-223 and miR-155-5p directly suppress CLDN8 and CLDN16, respectively, in a human cell line. Additionally, CLDN2 has been identified as a direct target of miR-16 and miR-125b, while being upregulated by miR-34a/c-5p and miR-29b-3p in human cell lines.^195^ This evidence indicates that miRNAs involved in barrier function are downregulated in IBD. Casado et al.^196^ demonstrated that fecal let-7b and miR-21 are critical molecules linked to colitis in both humans and mice. The let-7b significantly alters gut microbiota and enhances macrophage-associated pro-inflammatory cytokines, an effect contingent upon microbial presence. Conversely, miR-21 not only disrupts intestinal barrier integrity but also elevates myeloperoxidase and antimicrobial peptide secretion, resulting in dysbiosis of gut microbiota. This dysbiosis was notably ameliorated by anti-miRNAs, fostering healthier host – microbiota interactions within the intestinal lining.

## The gut microbiota and its metabolites influence IBD progression through chromatin remodeling

Few reports exist on the modulation of chromatin remodeling by gut microbiota and their metabolites that affect IBD. Jugder et al.^197^ reported that microbial-derived acetate induces chromatin remodeling within enteroendocrine cells, which co-regulates host metabolism and intestinal innate immunity through the conserved Tip60-steroid hormone axis in mammals. Kazakevych et al.^198^ discovered that Smarcad1 is a conserved chromatin remodeling factor and that the specific deletion of Smarcad1 in the intestinal epithelium of mice leads to significant effects. Targeted deletion of Smarcad1 in the murine intestinal epithelium led to colitis resistance, modifications in chromatin accessibility at numerous loci, and notable alterations in histone H3K9me3, indicating that highly conserved chromatin remodeling factors have a distinct function in antimicrobial defense.

## Conclusion and outlook

IBD is believed to result from the intricate interaction of several genetic predispositions and environmental triggers, resulting in gut microbiota dysbiosis and immune system hyperactivation. Although comprehensive genetic analyses of IBD exist, genetic factors cannot be evaluated in isolation from other influences to yield useful guidelines for the diagnosis or management of CD or UC. Currently recognized genetic factors contribute little to disease variability, which poses significant challenges for accurate diagnosis and treatment. In recent years, the importance of epigenetic modifications in mediating host – microbe and metabolite relationships in IBD has rapidly emerged. All genes and gene will undergo are subject to epigenetic modifications, which can explain the conversion from genotype to phenotype.^199^ This article presents an overview of the various types of epigenetic modifications in IBD, as derived from multiple sources, aiming to present a clearer perspective. While epigenetic modifications encompass various interdependent regulatory mechanisms that influence each other and collectively determine gene expression patterns and cellular functionality. Consequently, multi-omics analyses of the microbiome and host in IBD patients offer substantial potential for a comprehensive understanding of the host – microbe interactions that contribute to the pathogenesis of IBD.

The advent of next-generation sequencing and high-throughput technologies, such as metagenomics, metabolomics, and proteomics, has revolutionized our ability to systematically map the molecular components of these complex systems. Utilizing multi-omics approaches provides unprecedented opportunities to dissect host-microbe interactions in IBD.^200,201^ In recent years, large-scale integrative multi-omics analyses have rapidly developed. For instance, the 1000IBD Project recruited diverse types of patients IBD, conducting comprehensive and in-depth research across multiple domains, such as the association between dietary patterns and selective features of the gut microbiota,^79^ and the identification of environmental factors linked to IBD development.^202^ Robust bioinformatics tools can integrate genomic, epigenomic, transcriptomic, proteomic, metabolomic, and microbiomic data to construct a comprehensive molecular atlas of IBD. Combining metagenomics, metatranscriptomics, and metaproteomics can reveal various microbial activity molecules that may interact with the host immune system in IBD, providing insights into the broad and dynamic host-microbe interactions involved in IBD pathogenesis.^203^ These findings can be validated through in vitro and in vivo experiments, such as utilizing mouse models of colitis and patient-derived organoids and co-culture systems, to elucidate disease mechanisms. However, current research on the exposome, genome, microbiome, and immunome has largely been conducted in isolation, without considering the influence of other factors.

The heterogeneity of microbial composition in IBD may be due, in part, to the heterogeneity of host genotype and gut microbiota prior to pathogenesis. Although gut microbiota has been shown to modulate epigenetic modifications, the underlying mechanisms have not been fully elucidated, especially since the complexity of the epigenetic modifications themselves necessitates further research to fully understand their interactions and develop effective therapeutic interventions. Both host DNAm and gene expression showed a more pronounced inflammatory gradient than in gut microbiota, suggesting that epigenetic factors mediate the interaction between gut microbiota and host gene expression, with significant epigenome-wide differences observed between patients in the CD group and healthy controls.^154^ The host DNAm and gene expression in the IBD group were also more pronounced than in gut microbiota, suggesting that epigenetic factors mediate the interaction between gut microbiota and host gene expression.^204^ Currently, epigenetic modifications such as DNAm have been extensively studied, whereas research on other epigenetic modifications remains largely limited to animal models and cell cultures, which affects the authenticity of the data to some extent. Despite a wealth of data reported on susceptibility genes and epigenetic modifications, the clinical translation of these findings remains suboptimal.

Multi-omics, characterized by the joint analysis of proteomics, metabolomics, genomics, microbiomics, and epigenetics data, aims to achieve better patient stratification. This approach is expected to uncover new perspectives on IBD and other complex diseases associated with the microbiome, thereby informing the development of much-needed precision medicine strategies. Combination therapies targeting multiple histone-modifying enzymes or pathways may present a promising avenue for future research, and the development of more specific and targeted inhibitors, along with the identification of biomarkers, is important for IBD.

## Data Availability

Data sharing not applicable – no new data generated.

## Abbreviations

ACLY: ATP citrate lyase
AhR: Aryl hydrocarbon receptor
APT2: Acyl protein thioesterase 2
CD: Crohn’s Disease
CoA: Coenzyme A
CRC: Colorectal Cancer
DHHC: Asp-His-His-Cys
DNAm: DNA methylation
DNMT3a: DNA methyltransferases 3a
DNMTs: DNA methyltransferases
DSS: Dextran sodium sulfate
DUBs: Deubiquitinating enzymes
GWAS: Genome-wide association study
HAT: Histone acetyltransferases
HDAC: Histone Deacetylases
HDAC3: Histone deacetylase 3
IBD: Inflammatory Bowel Disease
IECs: Intestinal epithelial cells
LCA: Lithocholic acid
LncRNA: Long non-coding RNA
miRNA: MicroRNA
ω-3: omega-3
ω-6: omega-6
ncRNA: Non-coding RNA
OTUD: Ovarian tumor family deubiquitinase
PKM2: Pyruvate Kinase M2 Isoform
PTM: Post-translational modification
PUFAs: polyunsaturated fatty acids
SAM: S-adenosylmethionine
SCFAs: Short-chain fatty acids
ST6: ST6GalNac1
TLR: Toll-like receptors
Tregs: T-regulatory cells
UC: Ulcerative Colitis
Uhrf1: Ubiquitin-like with PHD and Ring Finger Domains 1
UMEs: ubiquitin-modifying enzymes
WGBS: Whole-Genome Bisulfite Sequencing
